# Supplementary material for: Functional Analysis of Antipsychotics in Human iPSC-Based Neural Progenitor 2D and 3D Schizophrenia Models
Source: Int J Mol Sci. 2025 May 7;26(9):4444. doi: 10.3390/ijms26094444 (PMC12072398; doi:10.3390/ijms26094444)
Supplement: Supplementary file 1 [file ijms-26-04444-s001.zip › ijms-3539355-supplementary.pdf]

Supplementary Materials: The following supporting information can be downloaded at: https://www.mdpi.com/article/10.3390/ijms26094444/s1, Figure S1: Scratch assays in different NPC cultures; Figure S2: Characterization of receptor expression based on mRNAseq data; Figure S3: Characterization of outgrowth of differentiating spheroids.

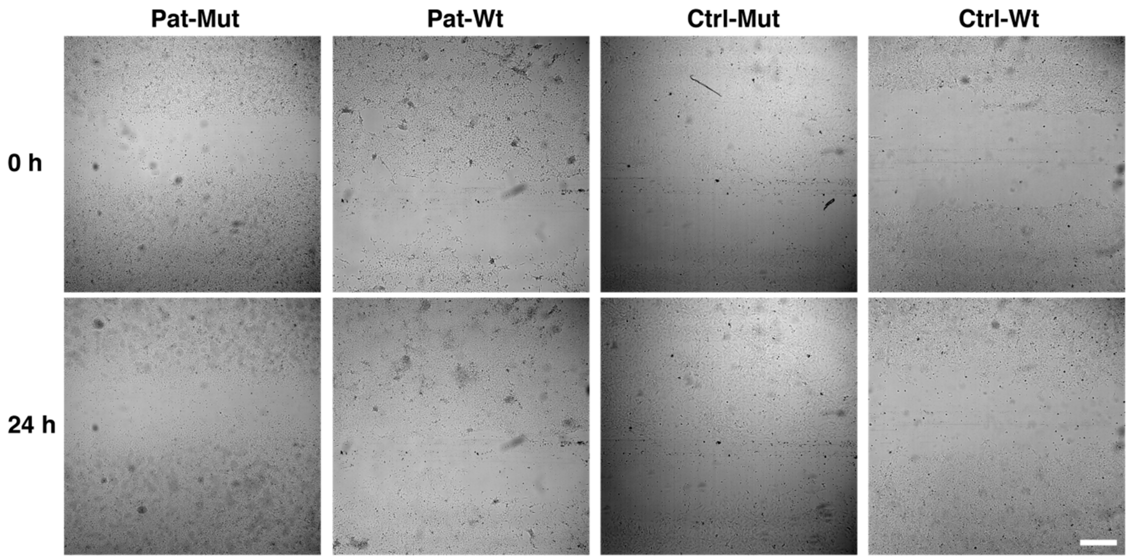

Supplementary Figure S1. Scratch assays in different NPC cultures. Representative pictures of scratch assays in different NPC cultures. The scale bar represents 500 µm.

	NPC				Neuron			
receptors (antipsychotics)	Pat-Mut	Pat-Wt	Ctrl-mut	Ctrl-wt	Pat-Mut	Pat-Wt	Ctrl-mut	Ctrl-wt
D1	0.28	6.78	0.10	1.53	4.80	4.65	3.37	1.71
D2 (HP,RP,AP)	0.07	0.23	0.47	0.10	0.71	0.07	0.13	0.69
D3 (HP,RP, AP)	0.00	0.07	0.59	0.22	2.10	0.32	1.00	1.39
D4 (HP,RP,AP)	1.04	2.23	2.89	1.40	2.98	2.17	2.33	1.80
D5	0.03	0.01	0.03	0.01	0.13	0.05	0.02	0.00
5 HT 1A, G protein-coupled (AP)	0.00	0.00	0.01	0.00	0.40	0.00	0.00	0.00
5 HT 1B, G protein-coupled	0.00	1.30	0.68	0.61	6.11	2.06	3.09	3.82
5 HT 1D, G protein-coupled	1.54	9.40	3.12	3.01	2.10	5.91	7.64	6.10
5 HT 1E, G protein-coupled	0.00	0.02	0.01	0.05	2.16	0.15	0.50	0.34
5 HT 1F, G protein-coupled	0.06	0.33	0.07	0.09	0.15	0.08	0.06	0.16
5 HT 2A, G protein-coupled (RP,AP)	0.51	1.76	0.15	0.42	8.07	7.19	6.34	3.85
5 HT 2B, G protein-coupled	0.50	0.51	0.74	0.44	0.58	0.14	0.44	0.18
5 HT 2C, G protein-coupled (AP)	0.09	0.07	0.05	0.02	4.56	1.22	2.09	0.44
5 HT 3A, ionotropic	0.10	0.03	0.03	0.00	0.46	0.05	1.09	0.77
5 HT 3B, ionotropic	0.76	0.49	1.36	0.11	15.88	0.56	7.83	4.45
5 HT 3C, ionotropic	0.00	0.00	0.05	0.00	0.28	0.00	0.18	0.00
5 HT 3D, ionotropic	0.00	0.00	0.01	0.01	0.00	0.02	0.06	0.00
5 HT 3E, ionotropic	0.00	0.01	0.07	0.00	0.14	0.00	0.03	0.05
5 HT 4, G protein-coupled	0.00	0.00	0.00	0.00	0.03	0.01	0.00	0.01
5 HT 5A, G protein-coupled	0.00	0.03	0.00	0.00	0.63	0.20	0.41	0.36
5 HT 6, G protein-coupled	0.07	0.08	0.42	0.12	1.50	0.34	1.34	0.70
5 HT 7, adenylate cyclase-coupled (RP,AP)	0.10	0.16	0.14	0.26	2.30	2.82	1.67	0.76

Supplementary Figure S2 Characterization of receptor expression based on mRNAseq data (a) mRNA expression of dopamine and serotonin receptors in NPCs and 6-week-old neurons. The antipsychotic that acts on the given receptor has been indicated in parentheses. The receptors on which antipsychotics act and are expressed in the in vitro system are highlighted in green. The expressions are indicated in RPKM (Reads Per Kilobase Million).

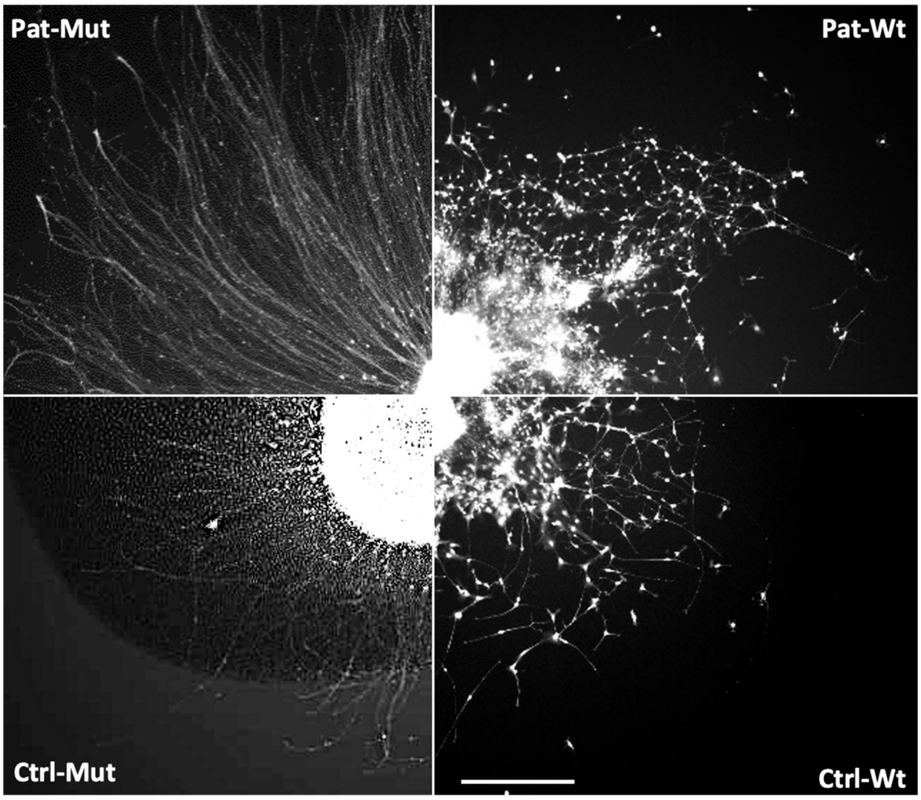

Supplementary Figure S3. Difference in the morphology of the outgrowth in differentiating spheroids. Representative and enlarged pictures of spheroids after 4 days of differentiation stained by Calcein-AM. The scale bar represents 500μm.

3D NPC cell number determination

Appendix A

Appendix A.1

The Macro code for 3D cell number analysis

```
// Step 1: Convert to 8-bit
run("8-bit");

// Step 2: Enhance Contrast with 0.35% saturation
run("Enhance Contrast", "saturated=0.35");

// Step 3: Threshold
setAutoThreshold("Default");
setOption("BlackBackground", true); // Ensure cells are highlighted (white foreground, black background)
run("Convert to Mask"); // Converts threshold to binary mask
run("Convert to Mask"); // Converts threshold to binary mask
```

```
// Step 4: Analyze Particles
// Size range can be adjusted to fit cell sizes, the best one was 1 for these pics
//Adjusting circularity does not improve
run("Analyze Particles...", "size=1-Infinity circularity=0.00-1.00 display summarize");appendix.
```
